# Real-World Experience With Givosiran in Acute Porphyrias: A Narrative Review and a Novel Hypothesis

**DOI:** 10.7759/cureus.104552

**Published:** 2026-03-02

**Authors:** Petro E Petrides

**Affiliations:** 1 Department of Porphyria Expertise, International Porphyria Network (IPNET), Hematology Oncology Center, Munich, DEU; 2 Department of Medicine, Ludwig Maximilian University of Munich, Munich, DEU

**Keywords:** acute porphyria elevate, asgp-receptor, bile acids, breakthrough attack, conundrum, envision trial, gall bladder, givosiran, pancreatitis, real-world experience

## Abstract

Real-world experience with givosiran has been accumulating after its approval for the treatment of patients with acute porphyria and chronic attacks in the United States and in Europe, respectively. Up to now, nearly as many patients from various countries have been treated after the registration of the drug and reported in real-world studies as treated in the randomized phase 3 ENVISION trial. Most reports confirm high drug efficiency, but some also report adverse effects (homocysteinemia, lipase elevation, and kidney function impairment). Moreover, many patients suffer from breakthrough attacks, which remain a conundrum. Since gallbladder epithelial cells also contain the asialoglycoprotein receptor, which is a prerequisite for the uptake of givosiran, it could be that the siRNA-induced depletion of haem leads to a disturbance of the function of the gallbladder. Therefore, we hypothesize a potential role of the gallbladder and the metabolism of bile acids in the development of porphyria and givosiran-resistant attacks, which has to be supported by clinical or experimental validation.

## Introduction and background

The acute porphyrias are a group of very rare diseases that are caused by genetic defects of enzymes of the haem (iron porphyrin) biosynthetic pathway. Depending upon which enzyme is involved, the major forms are called acute intermittent porphyria (AIP; porphobilinogen (PBG) deaminase), variegate porphyria (VP; protoporphyrinogen oxidase), and hereditary coproporphyria (HCP; coproporphyrinogen oxidase).

Under normal conditions, porphyrin biosynthesis is not impaired by the heterozygous enzyme deficiency. If, however, drugs induce the production of haem-containing CYP450 enzymes, haem biosynthesis becomes deregulated through the induction of 5-ALA-synthase 1 (the first and rate-limiting step) with the overproduction of 5-aminolevulinic acid (5-ALA) and PBG. The former metabolite is assumed to participate in causing acute abdominal pain, i.e., the classic symptom of porphyria attacks. The therapeutic regimen of choice is the infusion of haem arginate. A small proportion of patients experience chronic relapsing attacks that respond only weakly to treatment with haem arginate. For them, the development of givosiran has been a revolution: the drug belongs to the group of siRNAs, which act through binding to the mRNA of their target protein. By partially inhibiting 5-ALA synthase 1, givosiran slows down the production of 5-ALA and PBG and, through this, the precipitation of porphyria attacks [1].

Randomized clinical trials (RCTs) - in this case, the ENVISION trial [2] - are the gold standard for the evaluation of drug efficacy and safety. This prospective trial has been the prerequisite for the regulatory approval of givosiran (Givlaari®) in 2019 in the United States and in 2020 in Europe, and its subsequent incorporation into clinical practice.

In RCTs, strictly controlled inclusion and exclusion criteria, as well as the utilization of randomization procedures, minimize the impact of factors that potentially affect causal inferences. Hence, they provide more definitive conclusions and more reliable evidence. Nowadays, most RCTs are initiated by industry owing to high financial and organizational needs [3].

However, they also have limitations: stringent entry criteria may reduce the representativeness of the trial population to the target population, the standard trial interventions used may not be completely consistent with real-world clinical practice, the limited sample size and short follow-up time may lead to insufficient evaluation of rare adverse events. These limitations bring challenges during the extrapolation of RCT conclusions to real-world clinical practice.

The initial report of the ENVISION trial included an initial observation period of only six months [1]; when the 24-month open-label extension (OLE) period was published [4], it included our serendipitous observation on two patients with excessively elevated homocysteine levels - the analysis not being part of the study protocol [5]. This caused the retrospective analysis of all frozen patient samples, which revealed homocysteine elevation in all patients and led to the mandatory incorporation of vitamin B6 into the therapeutic regimen [6]. This example illustrates that laboratory adverse events cannot be detected when not specified in an RCT study protocol.

In this narrative review, I will summarize the real-world experience accumulated until now and propose a hypothesis to be validated in the clinic.

This paper is based on a plenary lecture given at the International Conference on Porphyrins and Porphyrias (ICPP) in Pamplona/Spain, in September 2024.

## Review

Real-world evidence (RWE)

Real-world data (RWD) or real-life data (RLD) are collected from various sources. RWE is the clinical evidence about the usage and potential benefits (effectiveness) and risks (safety) of a medical product derived from the analysis of RWD [7]. RWD includes more patients with numerous comorbidities. As the quality of RWD analyses reaches a high level, they help to improve the efficacy of the study design, generate new hypotheses, and provide information about drug adherence.

In contrast to prospective phase 3 trials, retrospective real-world case reports or case series (“aggregating case reports”) provide detailed information on patient illness, diagnostic problems, therapy, and treatment results over time in a retrospective fashion. Ideally, case reports are compiled following the CARE (CAse REport) guidelines for case reports (https://www.care-statement.org/). Consistent reporting would make it possible to use case reports in an aggregating fashion [8]. Hence, RCT and RWD studies are complementary.

Methods

PubMed and Google Scholar were regularly screened for new publications on givosiran and analyzed for drug efficacy and safety, as well as unexpected findings. The search started in 2021 until February 2026; the key Medical Subject Headings (MeSH) search terms were givosiran and ALA-synthase 1. All relevant publications found were included, i.e., no negative or conflicting studies were excluded.

This is an alternative to our real-world analysis on momelotinib, a novel JAK2 inhibitor for the treatment of myelofibrosis, where we send out questionnaires to participating centers [9].

Data were also collected through patient care in our international Porphyria Center [10]. In addition, personal exchanges with experts in the field were included.

RWE for givosiran

RWE on givosiran, which refers to its safety and effectiveness, in daily clinical practice in various patients with different comedications, comorbidities, or organ impairments, is necessary to supplement the experience obtained by the ENVISION trial.

Givosiran was registered in November 2019 in the United States (US) and in March 2020 in the European Union (EU) for the treatment of acute hepatic porphyria (AHP). All patients who have been treated since then are real-world patients. In some countries, patients could receive givosiran under a Temporary Authorization for Use (TAU) regimen (also called compassionate use), which allows the exceptional use of the drug.

The largest series of RWD, until September 2024, on givosiran were reported by the French group with 18 patients [11]: they set the pace for an individualization of the therapeutic regimen deciding to delay the injections in patients without acute symptoms and low haem precursor levels in urine and dividing their patients into two subgroups (injections every three months or less) due to a moderate or unstable decrease based on 5-ALA levels. As patients in the second subgroup had prolonged and stable precursor levels, they were only treated when their 5-ALA levels increased. This strategy did not compromise disease control but reduced the risk of side effects. Acute pancreatitis occurred in one patient, and all patients experienced an increase in their homocysteine levels. Early treatment was associated with a better treatment response. However, some of these patients experienced a decline in their renal function [12].

Two studies reported dramatic improvements in neurological symptoms after initiating givosiran treatment: one patient from Regensburg in Germany showed clearly improved tetraparesis and started to walk [13], whereas the other from Modena in Italy could walk unaided after being bound to a wheelchair for five years [14].

A study from Galveston in the US reported three patients [15]: Givosiran significantly reduced the frequency of attacks in one patient; however, occasional breakthrough attacks of the same symptoms occurred and were treated with haem. The authors discussed that “with a long history of attacks, other neurological mechanisms may become established that cause recurrent symptoms in the absence of porphyrin precursor elevations.” The other two patients also showed recurrent abdominal pain despite normalization of their laboratory values.

In one case from Winston-Salem in the US, recurrent symptoms also occurred after the normalization of biochemical values [16]. Givosiran was discontinued after decreased renal function in a report from Nagoya in Japan [17] and that from Winston-Salem in the US after liver injury [18].

In a series studying 11 patients from San Giovanni Rotondo in Italy, the authors distinguished between patients who showed a drastic improvement of their condition parallel to the reduction of PBG levels and those in whom abdominal pain with paraesthesia persisted for five months despite the improvement of their haem precursors levels [19]. In one patient from Bordeaux in France, homocysteine levels rose to 447 µmol/L. When the injection interval was extended to two to three months, and vitamin B6 was added to the treatment, givosiran treatment could be continued. However, 5-ALA-levels were normalized, while PBG levels remained relatively high (10-30 times the normal values), and the patient experienced no further attacks since starting givosiran. In another patient, it caused drug-induced liver injury (alanine transaminase (ALT) 559 U/L and aspartate aminotransferase (AST) 382 U/L) and hyperhomocysteinemia (87.9 µmol/l) [20].

In Seville, Spain, three patients responded well and were treated up to 46 months [21]. One report from Miami in the US indicated that givosiran may not be active in 5-ALA-dehydratase porphyria [22]. The only report of treating an affected child is from Springfield in the US [23].

In Galveston, a patient had a severe attack following a COVID-19 infection. After initial hematin treatment, a continuous givosiran therapy led to a nearly full recovery from a severe Guillain-Barré-like syndrome [24].

In total, 44 patients from various countries had been reported up to September 2024 (i.e., the date when the lecture was given, see Introduction) (Table 1).

**Table 1:** Real-world evidence for givosiran Real-world evidence (number of patients and outcomes) for givosiran in patients with acute intermittent porphyria (AIP), hereditary coproporphyria (HCP), and 5-aminolevulinic acid dehydratase (5-ALA-D) deficiency up to September 2024. The largest series originates from France.

No. of Patients	Outcomes	Reference
18	New individualized therapeutic approach	[11,12]
1	Improved neurological symptoms	[13]
1	Regained ambulation after being wheelchair-bound	[14]
3	Breakthrough attacks	[15]
1	Persistent abdominal pain despite normalized urinary 5-ALA levels	[16]
1 (HCP)	Sustained improvement >2 years after discontinuation of givosiran	[17]
1	No symptom relief; elevated transaminase levels	[18]
11	Heterogeneous clinical responses	[19]
1	Severe hyperhomocysteinemia treated with vitamin B6	[20]
3	Validation of individualized therapy	[21]
1 (5-ALA-D)	No clinical response	[22]
1	Resolution of abdominal and back pain (16-year-old boy)	[23]
1 (HCP)	Complete recovery from Guillain-Barré-like neurological syndrome	[24]

Since that time, additional reports from Nagoya in Japan (seven patients [25]) and Chemnitz in Germany (28 patients [26]) have been added. The Japanese study reported good efficacy and safety (surprisingly, only one patient had hyperhomocysteinemia). In the German real-world study with 28 patients (treated from 2018 to 2024, which means that ENVISION patients must have been included), givosiran could prevent severe attacks and reduce the chronic health burden. Two patients withdrew because of overwhelming fatigue. Again, patients with breakthrough attacks were observed who obtained haem arginate or glucose infusions. Importantly, about half of their cohort would have been ineligible for the ENVISION trial, having either a limited number of attacks or lacking prior prophylactic haem treatment.

The longest follow-up (48 months) analysis (phase 1/2 OLE) of 16 patients was reported from Stockholm in Sweden [27]: four patients had transient increases of lipase levels with no reported signs or symptoms of pancreatitis. One patient developed urticaria at the injection site, extending to her limbs, similar to what has been described by us [5].

From these publications with a very heterogeneous real life patient cohort, we can conclude that (1) individualization of givosiran dosing is beneficial for the patient, and quality of life of the patients improves dramatically (particularly, severe neurological symptoms can improve under givosiran treatment) and (2) nearly all investigators report that symptoms persist despite of normalized levels of urinary 5-ALA (breakthrough attacks) in some patients. No information is provided regarding possible confounding factors (e.g.type of PBG-deaminase mutation) or comorbidities.

The challenge of pancreatitis

In 2021, we reported a patient enrolled in the ENVISION study who developed excessive hyperhomocysteinemia (homocysteine level around 400 µmol/l) and a life-threatening necrotizing pancreatitis with lipase values of >2300 U/l [5]. This is in line with recent observations that pancreatitis is emerging as a rare complication of classical homocystinuria [28]. Increases in lipase and amylase levels (5/25), as well as acute pancreatitis (1/25), were also reported by others in up to 13% of patients [11,29]. Hence, an alert was included in the package insert of Givlaari® [30]: “Cases of acute pancreatitis, some severe, have been reported in Givlaari®-treated patients. Consider acute pancreatitis as a potential diagnosis in Givlaari® - treated patients with signs/symptoms of acute pancreatitis including acute upper abdominal pain, clinically significant elevation of pancreatic enzymes, and/or imaging findings of acute pancreatitis, to ensure appropriate management. Consider interruption and/or discontinuation of Givlaari® treatment for severe cases.”

Following up on our initial observation of a severe acute necrotizing pancreatitis in a givosiran-treated patient [5] and its possible origin (drug-induced, homocysteine-induced, or idiopathic), we are still intrigued by this complication: another patient reported in our Munich cohort [8] was offered givosiran after several sporadic attacks treated with haem arginate. Following the first injection (G1), his situation did not improve but got worse (persisting abdominal pain, nausea, loss of power) as seen by others [16]. Abdominal ultrasound showed two gallstones (1.6 and 1.0 cm in diameter) and no gallbladder sludge. At the same time, lipase levels increased to more than three times the upper limit of normal (from 26 to 62 U/mL), creatinine rose to 1.5 mg/dL, and homocysteine increased to 18.6 µmol/L despite vitamin B6 supplementation. The dosage of the next two injections (G2 and G3) was reduced by 50%, but his state did not change; thus, therapy was discontinued. His urine 5-ALA and PBG levels over time are shown in Table 2. Interestingly, the patient had no symptoms in 2018 and 2019 when secreting larger amounts of 5-ALA and PBG into the urine (an asymptomatic high secretor according to the international Delphi consensus) [31].

**Table 2:** Givosiran treatment Precursor levels of 5-ALA and PBG and symptoms of the reported patient. * below 6.40 mmol/mol creatinine ** below 2.24 mmol/mol creatinine (analysis by IPNET-Lab MVZ Karlsruhe) 5-ALA: 5-aminolevulinic acid; PBG: porphobilinogen

Date	12/18	02/19	06/21	04/22	03/23	11/23
5-ALA*	160	118	63	8.2	1.2	3.65
PBG**	81	121	70	16.2	1.8	13.4
Symptoms	no	no	no	yes	no	no
Givosiran	-	-	-	G1	G2	G3

The role of 5-ALA for acute attacks

These observations support the postulate that an increase of 5-ALA is necessary but not sufficient for the precipitation of an attack [32]. This is in line with a recent report from the US identifying 31% of asymptomatic high excreters [33] and an older study by Marsden and Rees [34], who had reported that urinary metabolites (including 5-ALA) remain elevated for many years after an acute attack.

In addition, there are other aspects of 5-ALA to consider: in medical practice, 5-ALA is used in combination with radiation to treat malignant and inflammatory diseases [35]. It is registered in the EU under the name Gliolan® [36] for this application. In Japan, it is freely available as a nutritional supplement [37]. Animal studies showed its preventive effect in type 2 diabetes mellitus [38]. Moreover, modulation and proteomic changes of the haem pathway were demonstrated after treatment with 5-ALA in cell culture experiments [39].

Additionally, more than 30 years ago, Mustajoki et al. infused 5-ALA in a male volunteer after a loading dose at a rate of 50-80 mg per hour for 92.5 hours. During the experiment, plasma 5-ALA and PBG concentrations rose to levels seen during acute attacks (9.11 µmol/L and 3-6 µmol/L, respectively) [40].

In tumour patients, the plasma 5-ALA levels were determined in a pharmacokinetic study after three different oral doses of 5-ALA (0.2 or 2.0 or 20 mg/kg body weight): peak levels were reached within 90 minutes and went up to a maximum of 8000 µg/L (60 µmol/L /135 µg = 1 µMol) without side effects [41].

All the mentioned applications do not cause a clinical picture resembling acute porphyria attacks. This makes it likely that an additional metabolite (“substance X”) is necessary to fully explain the precipitation of acute attacks.

Symptoms and causes of pancreatitis

Symptoms of pancreatitis are characterized by abdominal pain radiating to the back, which is similar to what porphyria patients can report during an attack. Causes for pancreatitis comprise gallstone migration, biliary sludge (40%), alcohol ingestion (30%), hypertriglyceridemia (2-7%), drugs (less than 5%), or other reasons (such as genetic, ERCP, infections, or hyperhomocysteinemia) [42].

Gallbladder abnormalities and acute pancreatitis

The severity of acute pancreatitis is often associated with gallbladder abnormalities (thickened wall, pericholecystic fluid, stones, and enlargement or dilatation of the bile duct) seen on MRI [43]. An abdominal ultrasound of our patient had shown two gallstones and no sludge. Control ultrasound examinations revealed an identical picture (no migration); thus, his symptoms were unlikely to be caused by the stones.

Uptake of givosiran into the hepatocyte

A prerequisite for givosiran uptake into the hepatocyte is the presence of a lectin receptor, the asialoglycoprotein receptor (ASGPR1), which consists of two subunits [44]. This transmembrane receptor is highly specific for N-acetylgalactosamine (GalNAc) and can deliver “cargos” containing this molecule into the liver.

**Figure 1:**
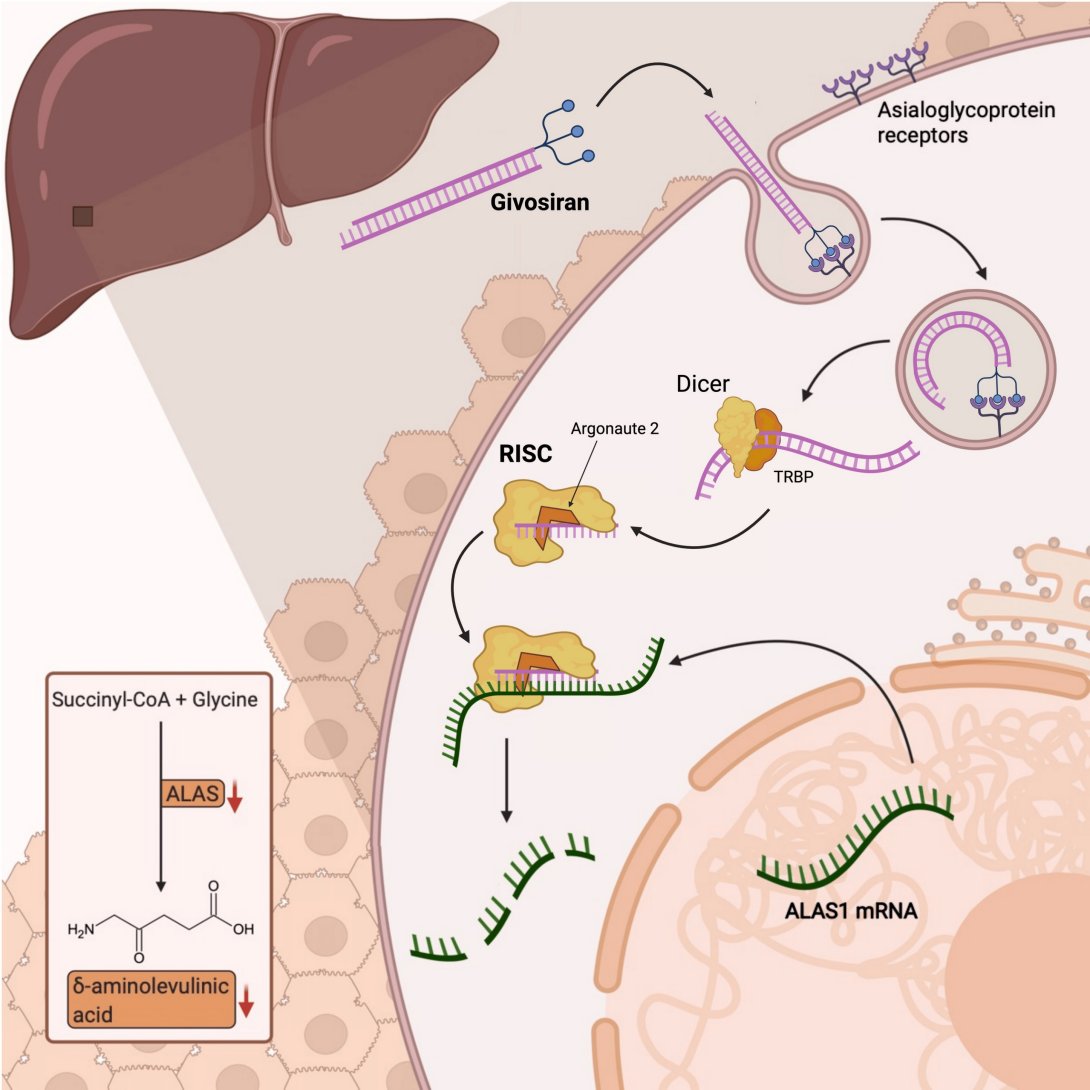
Givosiran uptake in the liver The binding of givosiran to the asialoglycoprotein receptor causes the formation of an endocytic vesicle. After delivery into the cytoplasm, the siRNA is processed by the endoribonuclease Dicer with the support of the RNA-binding cofactor transactivation response element RNA-binding protein (TRBP) and loaded by argonaute into the RNA-induced complex (RISC). The two strands of the siRNA get separated: the antisense binds to complementary sequences of the ALAS1-mRNA, with its consecutive partial inactivation of the enzyme and the consecutive decrease of 5-ALA and PBG. ALAS1: 5-aminolevulinic acid synthase 1; PBG: porphobilinogen; 5-ALA: 5-aminolevulinic acid Source: Reproduced with permission from the authors [45].

When givosiran is coupled through a linker to GalNAc, it will be taken up by the liver and released from the endosome within the hepatocyte (Figure 1) [45].

Unexpected expression of the asialoglycoprotein receptor in the gallbladder

**Figure 2:**
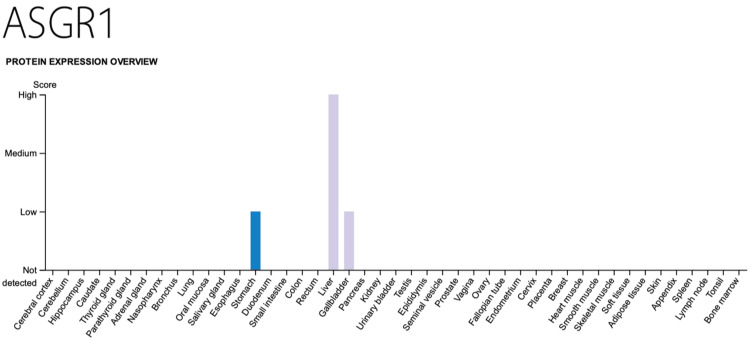
Asialoglycoprotein receptor Tissue distribution of the asialoglycoprotein receptor-1, which is also present in low concentrations in the gallbladder (and stomach). Source: Image obtained from The Human Protein Atlas [46], licensed under Creative Commons Attribution-ShareAlike 4.0 International (CC BY-SA 4.0).

The general assumption is that ASGPR1 is only expressed in the liver, making the uptake of givosiran tissue-specific. However, when the protein atlas (an open-access resource for human proteins) is consulted, AGSPR1-protein is also expressed in the gallbladder (Figure 2) [46].

This may link a side activity of givosiran to the gallbladder. One could hypothesize that givosiran also binds - though to a lesser extent - to the gallbladder epithelium. Since the biosynthesis of haem occurs in all tissues, givosiran could impair its synthesis in gallbladder epithelial cells. Depletion of bioavailable haem would then lead to mitochondrial dysfunction and impaired oxidative phosphorylation [47]. Moreover, haem-dependent gas sensing, gating of ion channels, or CYP450 enzymes could be influenced: at least two of such enzymes, CYP1A1 [48] and CYP5A [49], have been described in gallbladder specimens. Recent publications have indicated that AGSR1 is also found in dendritic and other cells [50].

Functions of the gallbladder

According to traditional thinking, the functions of the gallbladder include fluid transport across the epithelium and motor activity to expel the bile formed by the liver into the duodenum. Hence, it is assumed that the gallbladder is not indispensable for life: 650,000 cholecystectomies per year are carried out alone in the US [51]. But the gallbladder is not simply a storage container of hepatic bile: recent studies have shown that bile acids are not only detergents but also versatile nutrient hormones, which can influence metabolic processes [52]. Through changes in bile acid composition (through absorptive and secretory capacities) and bile acid flow, the gallbladder can govern metabolic processes (see below), i.e., it can be regarded as a metabolic orchestrator.

Metabolic regulation through bile acids

**Figure 3:**
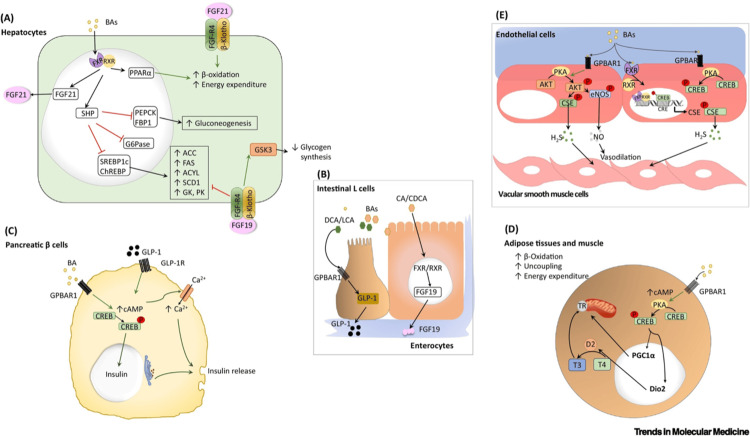
Bile acid signalling The multiple actions of bile acids on different organs: (A) Liver: Stimulation of FXR (see text) activates gluconeogenesis, 5-ALA-synthase (not shown), and ß-oxidation of fatty acids. It also releases FGF21. (B) Intestinal cells: Promotion of the release of GLP-1 and FGF-19. (C) Pancreas: Bile acids stimulate the release of insulin. (D) Adipose tissue: The prohormone T4 is converted into the active thyroid hormone T3. (E) Endothelial cells: Stimulation leads to the generation of NO and hydrogen sulfide. BAs: bile acids; FXR: farnesoid X receptor; 5-ALA: 5-aminolevulinic acid; GLP-1: glucagon-like peptide-1; FGF-19: fibroblast growth factor-19; NO: nitric oxide Source: Reproduced with permission from the authors [53].

Bile acids act as signaling molecules in various organs such as the liver, endocrine pancreas, enterocytes, endothelial cells, and adipose tissue (Figure 3) [53]. This may include regulation of intermediary metabolism (ß-oxidation, gluconeogenesis), release of cytokines (FGF 19, FGF 21) and hormones (insulin, GLP-1), hormone activation (thyroxin), or release of smooth vessel active substances (such as nitric oxide (NO)).

The activity of bile acids as signaling molecules is also connected to haem biosynthesis, which is mediated through their binding to the hepatic nuclear farnesoid X receptor (FXR), which activates 5-ALA-synthase-1 [54]. This has confirmed an earlier report [55]. Treatment with bile acids alleviates protoporphyrin accumulation in the liver of patients with erythropoietic protoporphyria [56].

Givosiran, CYP450 enzymes, and bile acid synthesis

**Figure 4:**
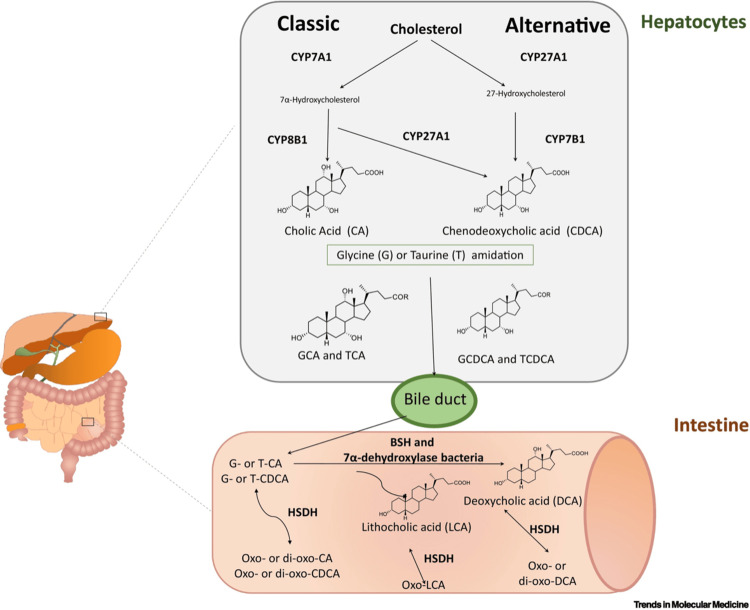
Bile acid synthesis and degradation Hepatic synthesis occurs on two pathways (classic and alternative) under the catalysis of CYP450 enzymes and degradation in the intestine. Both pathways generate primary bile acids in the liver. Amidation by conjugation with taurine (T) or glycine (G) leads to bile salts (before their secretion into the gut). These bacteria carry out two biotransformations: bile salt hydrolase (BSH), which deconjugates bile acids with the release of free bile acids (CA and CDCA). This is followed by the generation of secondary bile acids (DCA and LCA). In addition, hydroxysteroid-dehydrogenases (HSDHs) in bacteria can further generate several derivatives. CYP450: cytochrome P450; CA: cholic acid; CDCA: chenodeoxycholic acid; DCA: deoxycholic acid; LCA: lithocholic acid Source: Reproduced with permission from the authors [53].

Bile acids (cholic acid and chenodeoxycholic acid) are synthesized in two parallel pathways (with and without HO-C12 alpha) through CYP450 enzymes (Figure 4). Since givosiran inhibits cytochrome P450 more substantially than initially reported [57], bile acid synthesis in the liver may also be influenced by givosiran.

The conundrum

Some patients continue to have porphyria-like symptoms despite normalized urine 5-ALA values, as seen in our patient and reported by several other authors (see Table 1). This may be explained by the fact that givosiran induces gallbladder and bile acid dysfunction (synthesis and enterohepatic circulation) with a potential effect on the pancreas, intestine, or endothelium. This has to be proven by experimental and clinical validation. Herbert Bonkovsky and his colleagues have called porphyria-like symptoms despite normalized urine 5-ALA values “an ongoing clinical conundrum” [16]. The origin of the word conundrum, meaning “anything that puzzles,” is itself a conundrum. Although it resembles Latin, conundrum likely belongs to the same family of pseudo-Latin terms as hocus pocus. The earliest clue to the conundrum’s origins is a 1645 text that connects the term to Oxford University and appears to define it as “pun or wordplay“ [58].

Real-world registries

Real-world registries such as the global ELEVATE (NCT 04883905) with 28 sites may help to solve this conundrum [59]. They can also test new hypotheses as presented here, which incorporate a role of the gallbladder and bile acids in the pathogenesis of porphyrias and givosiran treatment.

Metabolomic analyses in acute porphyria patients

Recently, Lefebvre et al. have performed a non-targeted metabolomic analysis on the urine of patients with overt AIP and asymptomatic carriers [60]. Bile acids showed significant concentration differences between the two phenotypic groups. Dysregulation of bile acid metabolism revealed an imbalance in favor of hydrophobic bile acids (chenodesoxycholic acid (see Figure 4) and the secondary bile acid lithocholic acid) with changes in conjugation patterns (tauro-, glyco-, or sulfoconjugation), which was more pronounced in the severe phenotype that may connect to our hypothesis.

## Conclusions

Randomized phase 3 trials are the basis for establishing the efficacy and safety of a novel drug. The ENVISION trial has convincingly proven these prerequisites, which led to the approval of givosiran. Thereafter, real-world experiences in various countries with a heterogeneous cohort of patients began to accumulate, which also showed that some patients continue to have symptoms despite normalized PBG and 5-ALA values, which has been called a conundrum. Givosiran may also target the gallbladder because of the presence of the ASGR-receptor. The potential depletion of haem in this organ may disturb the metabolism of bile acids, which also act as signaling molecules in the gastrointestinal tract. This is in line with recent non-targeted metabolomic analyses on the urine of patients with overt AIP and asymptomatic carriers. Bile acids showed significant concentration differences between the two phenotypic groups, which caused a dysregulation of bile acid metabolism.
